# The economic impact of diabetes through lost labour force participation on individuals and government: evidence from a microsimulation model

**DOI:** 10.1186/1471-2458-14-220

**Published:** 2014-03-04

**Authors:** Deborah Schofield, Michelle M Cunich, Rupendra N Shrestha, Megan E Passey, Lennert Veerman, Emily J Callander, Simon J Kelly, Robert Tanton

**Affiliations:** 1 NHMRC Clinical Trials Centre and School of Public Health, Sydney Medical School, The University of Sydney, Sydney, Australia; 2 NHMRC Clinical Trials Centre, Sydney Medical School, The University of Sydney, Sydney, Australia; 3 University Centre for Rural Health – North Coast, School of Public Health, University of Sydney, Lismore, NSW, Australia; 4 School of Population Health, University of Queensland, Brisbane, Queensland, Australia; 5 National Centre for Social and Economic Modelling, University of Canberra, Canberra, ACT, Australia

**Keywords:** Diabetes, Chronic disease, Labour force participation, Economic costs, Microsimulation modelling, Income, Taxation, Government welfare payments, GDP

## Abstract

**Background:**

Diabetes is a costly and debilitating disease. The aim of the study is to quantify the individual and national costs of diabetes resulting from people retiring early because of this disease, including lost income; lost income taxation, increased government welfare payments; and reductions in GDP.

**Methods:**

A purpose-built microsimulation model, Health&WealthMOD2030, was used to estimate the economic costs of early retirement due to diabetes. The study included all Australians aged 45–64 years in 2010 based on Australian Bureau of Statistics’ *Surveys of Disability, Ageing and Carers*. A multiple regression model was used to identify significant differences in income, government welfare payments and taxation liabilities between people out of the labour force because of their diabetes and those employed full time with no chronic health condition.

**Results:**

The median annual income of people who retired early because of their diabetes was significantly lower (AU$11 784) compared to those employed full time without a chronic health condition who received almost five times more income. At the national level, there was a loss of AU$384 million in individual earnings by those with diabetes, an extra AU$4 million spent in government welfare payments, a loss of AU$56 million in taxation revenue, and a loss of AU$1 324 million in GDP in 2010: all attributable to diabetes through its impact on labour force participation. Sensitivity analysis was used to assess the impact of different diabetes prevalence rates on estimates of lost income, lost income taxation, increased government welfare payments, and reduced GDP.

**Conclusions:**

Individuals bear the cost of lost income in addition to the burden of the disease. The Government endures the impacts of lost productivity and income taxation revenue, as well as spending more in welfare payments. These national costs are in addition to the Government’s direct healthcare costs.

## Background

Diabetes affects millions of individuals worldwide, and the number of sufferers is expected to increase [1]. Indeed, the global burden of diabetes (measured in Disability Adjusted Life Years or DALYs) has already increased by 43.1% over the last 20 years [2]. Globally, there has been a significant increase in the number of deaths due to diabetes. The Global Burden of Disease Study 2010 reports 1.3 million deaths due to diabetes worldwide in 2010 which is twice as many as in 1990 [3]. For these reasons, diabetes has been labelled “one of the most common, severe, and costly diseases” [4].

In Australia diabetes is a ‘national health priority area’ in recognition of the substantial individual and government costs of the disease [5]. In 2007 about 4% of the Australian population has been diagnosed with diabetes [6]. In 2004 it was estimated that almost AU$1 billion had been spent on direct health system costs for diabetes in Australia, and that the cost of treating diabetes is expected to rise by over 400% from 2003 to 2033 [7,8].

While these direct costs are large, there is evidence internationally that the indirect costs of diabetes are greater than the direct costs [4]. These indirect costs are mostly attributed to lost productivity, with diabetes affecting an individual’s ability to engage in employment and hence reducing their income [9]. Indeed, a recent study of older working-age Australians found that diabetes was the eighth most common reason for not being in the labour force [10].

With the health burden of the condition being so large, the cost of diabetes on economies worldwide is correspondingly significant. In Europe, the extra healthcare and labour productivity costs associated with diabetes have become pressing challenges for governments. Consequently, several European stakeholders (such as The Fit for Work Europe Coalition) have put forward the case for counting work as a relevant outcome measure in health investment decisions, especially decisions involving patients with long-term chronic illnesses [11].

With the ageing of the global population, there is increasing focus on the need to retain older workers [12]. In Australia, 38% of workers aged 45–64 years who have diabetes are not in the labour force, representing a pool of people who may have participated if they did not have this disease [10]. Australia, like other developed countries, will need to maximise the labour force participation of its older workers to maintain adequate government revenue to fund health carefor an ageing population [13]. This makes the impact of diabetes on the labour force participation of older workers a vital issue.

This study examines the costs of early retirement due to diabetes. It estimates, for Australians aged 45–64 years, the amount of lost income to individuals, lost income taxation revenue to government, increased government welfare payments and lost Gross Domestic Product (GDP) due to these individuals leaving the labour force prematurely because of their diabetes. It also estimates the difference in these values between people who have retired early due to diabetes and those in full and part time employment with no chronic health condition. Most of this burden is, in principle, preventable. Diabetes Australia (2013) estimates that 89% of people aged 40–59 years with diabetes in Australia have Type 2 diabetes [14], which is associated with unhealthy lifestyles (unmanaged cholesterol, blood pressure; smoking; unhealthy food choices; sedentary lifestyle) and obesity [15]. We undertook sensitivity analysis where our estimates of lost income, lost income taxation, extra government welfare payments and lost GDP were reduced in line with this prevalence rate to estimate the potentially preventable component of these losses.

### Methods

#### Data

We used Health&WealthMOD2030, a microsimulation model of health, disability and labour force participation, to analyse the impact of diabetes on labour force participation, individual income, and government income (revenue) and spending. Health & WealthMOD2030 was purposely designed to evaluate the economic consequences of ill health on the labour force participation of Australians aged 45–64 years.

The base population of Health & WealthMOD2030 was unit record data for individuals aged 45–64 years drawn from the pooled data from the 2003 and 2009 *Surveys of Disability, Ageing and Carers* (SDACs) conducted by the Australian Bureau of Statistics (ABS) [16,17]. These nationally representative (Australian) household survey data provide information on individual characteristics (such as age, sex, family type, and state of residence), socioeconomic characteristics (such as highest level of education, income, type of home ownership, and government welfare payments), labour market characteristics (such as labour force participation, employment restrictions, and retirement), and health and disability characteristics (such as chronic conditions, health status, type and degree of disability, support and care requirements) for each person in the household. Australian Universities are provided with free access to the ABS unit record data that underpins this study under the Vice Chancellor’s Agreement.

Respondents in the SDACs reported what their main and other chronic health conditions were, and their responses were classified by the ABS [16,17]. In this study, respondents were considered to be out of the labour force due to diabetes if they stated they were out of the labour force due to illness and listed diabetes as their main chronic health condition. Like we did here, previous studies [10,18,19] have extracted data from national household survey data on people who have left the labour force because of their ill health or disability and used “main chronic condition” data to identify the disease having the largest (disabling) impact on individuals.

Whilst we cannot identify the type of diabetes people have from the SDAC data (as it is a single option in the survey), we note that the type of diabetes these people have is likely to be Type 2 diabetes based on the age group of study participants (Diabetes Australia reports that 89% of the diabetes prevalence of people aged 40–59 years in Australia is Type 2 diabetes) [14]. The 2003 and 2009 SDACs data were reweighted to reflect the profile of the 2010 Australian population aged 45–64 years using a reweighting algorithm *GREGWT*. The mathematical technique is described in Singh and Mohl (1996) [20] and an implementation of the same algorithm for small area estimation is described in Tanton et al. (2011) [21]. The ABS developed this algorithm, and related software, and commonly uses it to reweight their survey data. This reweighting procedure was used to account for the expected changes in disability and illness, demographics, labour force participation and other features of the population that occurred between the years for which we have data (2003 and 2009) and 2010.

The SDACs included only limited economic data, which were presented in ranges. Thus more detailed information on income, welfare payments and individual income taxes (and wealth) were derived from a separate microsimulation model called the Australian Population and Policy Simulation Model (APPSIM). APPSIM is a dynamic population microsimulation model that was developed to provide a snapshot output of demographic and economic characteristics (such as income and government welfare payments) of the Australian population in each year. It is developed and maintained by the National Centre for Social and Economic Modelling (NATSEM; http://www.natsem.canberra.edu.au). Detailed economic information from the APPSIM snapshot output for the year 2010 were imputed onto the base population of Health & WealthMOD2030 by identifying persons with similar characteristics on the concatenated SDACs and APPSIM. We imputed this more detailed income and wealth information onto Health&WealthMOD2030 using a process commonly used in microsimulation modelling called synthetic matching [22]. Ten variables that were common to both datasets and strongly related to income were chosen as matching variables for synthetic matching: labour force status (4 groups: employed full time, employed part time, unemployed, not in the labour force), income unit type (4 groups: married couple with dependents, married couple only, one parent with dependents, one person), income quintile (5 groups: income quintiles 1^st^-5^th^), receiving age pension (2 groups: yes or no), receiving disability support pension (2 groups: yes or no), sex (2 groups: male or female), age group (4 groups: 45–49 years, 50–54 years, 55–59 years, 60–64 years), hours worked per week (5 groups: 1–15 hours, 26–24 hours, 25–34 hours, 35–40 hours, 41-plus hours), highest educational qualification (2 groups: university or non-university) and home ownership (2 groups: yes or no).

#### Statistical analysis

Descriptive analysis was undertaken to establish the mean and median annual income, income taxation payments, and government welfare payments received by individuals employed full time, part time, and not in the labour force because of diabetes, expressed in 2010 Australian dollars.

A multiple regression model of the log of annual income was used to assess the differences between annual incomes of people in employment (full time) who reported they did not have a chronic health condition and people not in the labour force who reported they had left because of their illness and nominated diabetes as their main chronic condition. Similar models were estimated for the logs of annual welfare payments and tax liability. Each regression model was adjusted for the (individual) effects of age, sex and education. Modelling was undertaken on log-transformed data to satisfy the assumptions of the linear regression model. Diagnostic tests confirmed that these assumptions were satisfied.

The impact of diabetes on national GDP was estimated using the Commonwealth Treasury’s GDP formula:

$$\mathrm{GDP}=\left(\mathrm{GDP}/H\right)\times \left(H/\mathrm{EMP}\right)\times \left(\mathrm{EMP}/\mathrm{LF}\right)\times \left(\mathrm{LF}/\mathrm{Pop}15^{+}\right)\mathrm{\times Pop}15^{+}$$

where GDP = Gross Domestic Product; H = total hours worked; EMP = total number of persons employed; LF = size of the labour force; and Pop15^+^ = population aged 15 years and over [13].

All analyses were conducted in SAS V9.3 (SAS Institute Inc., Cary, NC, USA). All statistical tests were two sided with the significance level set at 5%.

#### Sensitivity analysis

Initial data extraction from the 2003 and 2009 SDACs was of people aged 45–64 years who reported they had left the labour force because of ill health and that their main chronic condition was diabetes.

Diabetes Australia reports that 89% of people aged 40–59 years with diabetes have Type 2 diabetes in Australia [14]. We conducted sensitivity analysis using this proportion of people with Type 2 diabetes to represent the proportion of people with the disease who could be expected to remain in the labour force if the disease were prevented. These estimates of the economic costs of diabetes represent the potentially preventable component of the overall losses.

## Results

Amongst people surveyed in the SDACs aged 45–64 years, 345 were employed full time with diabetes; 105 were employed part time with diabetes; 46 people were out of the labour force because of diabetes; 6 606 were employed full time with no chronic health condition, and 2 373 were employed part time with no chronic health condition. After weighting these data to 2010, these survey records corresponded to 88 900 employed full time with diabetes, 25 330 employed part time with diabetes, 11 300 individuals out of the labour force because of diabetes, 1 413 500 employed full time with no chronic health condition, and 467 800 employed part time with no chronic health condition, in the age group 45–64 years within the Australian population.

Of the people who reported they left the labour force due to diabetes, 13% reported having diabetes only (i.e. 1 chronic condition), 11% reported 2 chronic conditions, 26% reported 3 chronic conditions, and 50% reported 4 or more chronic conditions. Common chronic conditions for people out of the labour force due to diabetes were hypertension, heart disease, stroke and high cholesterol, which are associated with Type 2 diabetes (http://www.diabetesaustralia.com.au/Understanding-Diabetes/What-is-Diabetes/Type-2-Diabetes/).

While those who worked full time and reported diabetes as their main condition had lower incomes than those who worked full time and had no chronic health condition, there was no significant difference between the two groups.

Those who were out of the labour force as a result of their diabetes had a median annual income (income derived from all sources including welfare payments) of $11 784. This is approximately half of the median annual income of workers employed part time with no chronic condition ($21 257 per year) and approximately one fifth of the annual income of those employed full time with no chronic condition, $54 795 (Table 1). People out of the labour force because of their diabetes also had a lower median income compared to those with diabetes who were working either part time ($26 032) or full time ($52 655). Of their entire annual income, those people out of the labour force because of their diabetes received a median of $986 per year in government welfare payments, whereas those in the labour force received none as a median value. Those out of the labour force because of their diabetes paid effectively no (median) tax per year whereas those employed in full time work without a chronic health condition paid a median amount of $9 814 per year in tax.

**Table 1:** Average and median* annual income, government welfare payments^**# **^**and tax liability by labour force status for the Australian population aged 45–64 years, 2010**

**Labour force status**	**N**	**Annual income (AU$) received by individuals**			**Annual welfare income (AU$) received by individuals**			**Annual tax (includes Medicare levy) (AU$) paid by individuals**		
	**Surveyed weighted records population**	**Mean**	**SD**	**Median**	**Mean**	**SD**	**Median**	**Mean**	**SD**	**Median**
Employed full time, no chronic health condition	6 606 1 413 511	63 190	35 182	54 795	593	2 462	0	12 894	11 314	9 814
Employed part time, no chronic health condition	2 373 467 796	29 068	30 037	21 257	1 960	4 351	0	3 895	8 391	865
Employed full time, with diabetes	345 88 889	60 389	30 117	52 655	305	1 728	0	11 874	10 054	8 937
Employed part time, with diabetes	105 25 329	30 857	25 743	26 032	2 377	5 048	0	3 966	7 691	1 366
Not in labour force due to diabetes	46 11 310	13 900	16 727	11 784	6 048	7 296	986	1 040	4 068	0

*All results given in 2010 Australian dollars (AU).

#Government welfare payment in APPSIM (our data source for financial measures) includes:

•Aged pension – this payment provides income support and access to a range of concessions for eligible older Australians (aged 64–65 years and older).

•Disability support pension – financial support for people who have a physical, intellectual or psychiatric condition that prevents them from working or who are permanently blind.

•Newstart allowance – financial assistance for people who are looking for work.

•Youth allowance – financial assistance for people aged 16–24 years who are studying full time, undertaking a full time Australian apprenticeship, training or looking for work.

•Carer payment – an income support payment for people who personally provide constant care in the home of someone with a severe disability, illness or who is frail aged.

•Family tax benefits – a two part payment that assists with the cost of raising children (A and B).

Information on these payments can be found at: http://www.humanservices.gov.au/customer/services.

Compared to those in full time work and without a chronic health condition (and adjusted for age, sex and education), people out of the labour force due to diabetes received approximately 88% less in total income per year, on average (Table 2). This group also paid a significantly smaller amount in taxes per year, and received a significantly larger amount in government welfare payments per year than those employed without a chronic condition or employed with diabetes.

**Table 2:** Differences in average weekly income, welfare payments and tax liability between labour force status, adjusted for age group, sex and education, for the Australian population aged 45–64 years, 2010

**Labour force status**	**Income**			**Welfare income**			**Tax liability (includes Medicare levy)**		
	**% difference**	**95% CI**	**p-value**	**% difference**	**95% CI**	**p-value**	**% difference**	**95% CI**	**p-value**
Employed full time, no chronic health condition		*Reference*			*Reference*			*Reference*	
Employed part time, no chronic health condition	−56.3	(−58.7, −53.7)	<.0001	71.9	(42.5,107.2)	<0.0001	−96.1	(−97.0,-95.0)	<.0001
Employed full time, with diabetes	−6.5	(−13.2,0.7)	0.0746	24.9	(3.4,50.8)	0.0208	−24.6	(−42.0,-1.9)	0.0352
Employed part time, with diabetes	−52.4	(−60.2,-43.1)	<.0001	112.3	(0.4,348.7)	0.0488	−92.6	(−96.8,-83.0)	<.0001
Not in labour force due to diabetes	−88.3	(−95.6, −69.3)	<.0001	3 819.8	(793.5, 17 095)	<0.0001	−99.8	(−100.0, −99.2)	<.0001

The national impact of diabetes when it causes early retirement is $383.9 million in lost income earnings by those with diabetes, $56.4 million in lost income taxation revenue, and an extra $3.5 million in government welfare payments per year (Table 3) assuming that otherwise those with diabetes would have the same labour force participation rate and full time and part time employment rates as people without a chronic health condition. As a result of the (national) labour force losing 11 310 workers early because of diabetes, there was a loss of $1 324 million in GDP in 2010.

**Table 3:** National impact of diabetes (adjusted for age, sex and education) for the Australian population aged 45–64 years, 2010

	**Income (million AU$)**	**Transfer payments (million AU$)**	**Taxation revenue (million AU$)**
Not in labour force due to diabetes (n = 11 310)	383.9	3.5	56.4
Not in labour force due to preventable form of diabetes (type 2 diabetes; n = 10 066)	341.7	3.1	50.2

Note: Based on the differences between persons not in the labour force due to diabetes and the weighted average of persons employed full time and part time with no chronic health condition.

After taking into account the Type 2 diabetes prevalence rate for people aged 40–59 years in Australia [14], the number of people out of the labour force with this potentially preventable form of the disease was 10 066. The national impacts were $341.7 million in lost income earnings by those with diabetes, $50.2 million in lost income taxation revenue, and an additional $3.1 million in government welfare payments in 2010 (Table 3). As a consequence of losing these workers prematurely because of their (Type 2) diabetes, there was a loss of $1 179 million in GDP in 2010.

## Discussion

It is evident that diabetes is a costly disease. Diabetes comprises a substantial amount of government health funding, at AU$812 million in 2004-05 [8]. The majority of this funding is spent on hospital stays and prescription pharmaceuticals, followed closely by non-hospital medical services. In addition, the indirect costs of diabetes are considerable at the individual and national levels. People aged 45–64 years who left the labour force early because of diabetes had a median total annual income of only $11 784 whereas those working full time without a chronic health condition earned approximately five times this amount. These early retirees with diabetes also received a lower median income compared to those in the labour force with diabetes. The national impact of diabetes through losing 11 300 people aged 45–64 years from the labour force because of their diabetes was estimated to be $384 million in lost income earnings by those with diabetes, $56 million in lost income taxation revenue, $4 million in additional government welfare payments, and overall $1 324 million in lost GDP in 2010. After taking into account the type of diabetes that is potentially preventable for these workers [14], the national impacts were $341.7 million in lost income earnings by those with diabetes, $50.2 million in lost income taxation revenue, and an extra $3.1 million in government welfare payments in 2010. As a consequence of losing 10 066 workers prematurely because of Type 2 diabetes, there was a loss of $1 179 million in GDP in 2010.

A number of studies have demonstrated that diabetes has a significant impact on the ability of individuals to participate in the labour force. In Canada, for example, diabetics with complications were twice as likely to be out of the labour force compared to non-diabetics in the working-age population [9]. Similar findings have been presented in studies based on the United States and Australian working-age populations [5,23,24]. Complementing these studies on the direct impact of diabetes on labour force participation, others have focused on the indirect costs of diabetes through lost labour productivity and work absences of sufferers [4,24]. These studies support the findings reported in this paper, concluding that diabetes affects individual income through preventing labour force participation.

There are several limitations of the analysis. Firstly, the analysis is limited to people aged 45–64 years who reported that they are no longer in work as a result of having diabetes. It does not include other forms of limited labour force participation such as absenteeism (taking time off work while sick) and presenteeism (attending work while sick resulting in reduced productivity at work), nor does it estimate the value of lost GDP through early mortality, or the economic impact of carers having to give up work. Although the study did not set out to cover these areas, an estimate of the wider economic impact of diabetes through lost labour force participation would most likely result in even higher indirect economic impacts.

Secondly, the age range of study participants is limited to 45–64 years, as this is the age range which experiences the largest economic impact because of Type 2 diabetes prevalence and plans for retirement. Latest data shows that Type 2 diabetes affects 7% of people with diabetes aged 0–20 years, 54% of people with diabetes aged 21–39 years, 89% of people with diabetes aged 40–59 years, and 94% of people with diabetes aged 60 years or older [14]. Data for people under 45 years was not analysed because the paper sought to evaluate the costs associated with the age group with the highest proportion of the preventable form of diabetes who, in turn, would incur the largest costs of the disease through premature exit from the labour force.

Lastly, this study takes the human capital approach to valuing productivity costs (i.e. the patient’s perspective) and thus counts any hour not worked as an hour lost and values these hours. It does not consider potential ‘friction periods’ – the time in which a replacement employee may be found. The friction method takes an employer’s perspective and only measures as lost those hours not worked until another employee takes over the work of the person who is ill [25-27]. The main difference in outcomes of the two approaches stems from the different time horizon used to measure and value productivity costs. The human capital approach, by considering every hour not worked as an hour lost perhaps until the individual reaches the age of retirement, tends to lead to higher costs. Thus the human capital approach may overestimate productivity losses. But the friction cost approach has also been criticised as is values lost labour during a short time period – the friction period – which is a time assumed until the vacant job is filled (usually 3–6 months) [26,27]. Consequently, the friction cost approach may underestimate productivity losses. Limited empirical evidence is available on the differences in outcomes derived from these approaches; but see van den Hout (2010) [26] for a comparison of outcomes in relation to rheumatoid arthritis.

In our study, we use the human capital approach which represents the perspective of the person who has become too ill to work, but to be conservative only report costs over a 12 month period. We also reported on government impacts in terms of welfare payments and lost taxation revenue as well as national GDP impacts. This choice was made because it seemed to best reflect the economic environment in Australia. In Australia, many vacant positions are not filled by people who are unemployed, but are more likely to be filled by someone moving from another position, thus creating another job vacancy. This is partly due to the need to find an appropriate skills match between the position and the applicant and barriers to labour mobility partly caused by Australia being a large country [28]. For example, there have been labour shortages in mining, however these positions are usually in remote areas and families with young children or whose partner is in employment may not be able to move. This results in a relatively long duration between unemployment and finding another job. In Australia, someone who is unemployed and on a government unemployment benefit (called Newstart in Australia), on average, has a little over three years before they find a new position and are no longer eligible for an unemployment benefit [29]. Further, when someone leaves the labour force too ill to work, they may become eligible for a Disability Support Pension, which is about 50% higher than the unemployment benefit, thus the welfare costs to government of a person too ill to work are higher than someone who is simply unemployed. (http://www.humanservices.gov.au/customer/enablers/centrelink/disability-support-pension/payment-rates)[30]. In terms of GDP, if someone unemployed were to fill a newly created position this would contribute to GDP growth, whereas filling a vacancy created by someone leaving the workforce permanently when they become chronically ill generates no growth.

For these reasons we felt that for this study, the friction cost method, which assumes that 3–6 months after someone leaves the labour force someone else will have filled the position, and thus the aggregate impact after 3–6 months is zero [26] would underestimate the effects of leaving the workforce due to ill health not only for the person themselves, who are faced with an ongoing loss of income, but also the impacts on unemployment benefits and GDP which are longer in duration in Australia than the 3–6 month provision in the friction cost method.

Interventions that increase the labour force participation rate of people with chronic conditions (such as diabetes) are likely to yield benefits to both individuals and government. Several studies have demonstrated that pharmacological (such as metformin) and lifestyle interventions to prevent or delay the development of Type 2 diabetes in high risk individuals are effective and cost-effective [31-33], and have the potential to increase the labour force participation rate of older workers with this form of diabetes which, in turn, may reduce income losses [34].

Reduced income amongst those who retire early due to diabetes may also lead to inadequate (personal) finance for future health care needs [35]. One study in the United States found a high correlation between chronic illness and financial stress, with one quarter of bankruptcies attributable to chronic illness [35]. This may reflect families being ill-prepared to deal with the financial implications of caring for a family member with a long-term health condition. In Australia, older workers who retire early due to diabetes have a value of total wealth that is 89.6% lower (includes savings, superannuation, and property) than that of older full time workers with no health condition [18], leaving them with minimal savings to cover their health care costs. However, universal health coverage in Australia implies reduced ability to pay from reduced savings is somewhat moderated (although 30% of health care costs in Australia are private coming from individuals, private health insurance and other non-government sources [36]). Nonetheless, patients without savings for private care often face long waiting times for appropriate care in the public system. Type 2 diabetes is increasing in prevalence worldwide. It is also becoming more common amongst the working-age population, particularly those aged 45–64 years [23]. Consequently, the impact of this form of diabetes on labour force participation and the flow-on national impacts are likely to become greater over time. To help reduce the costly outcomes of premature retirement because of diabetes, investment in prevention (Type 2 diabetes) seems desirable. This aligns with the health platform of the current Australian Government which recognises that prevention of chronic diseases can help increase labour force participation. It has been acknowledged that prevention will not only improve the health of the general population but help to maintain economic growth by sustaining human resources in production [13,37], with the added advantage of helping Government to ensure that future revenues will be sufficient to fund the health care of its ageing population [13].

## Conclusions

Individuals incur significant costs from lost income due to diabetes as well as the burden of the disease itself. The Government incurs the impacts of lost labour productivity and income taxation revenue, and increased expenditure on welfare payments in addition to direct healthcare costs. These financial costs provide further support for the importance of preventing this disabling condition.

## Competing interests

The authors declare that they have no competing interests.

## Authors’ contributions

DS conceived the research question, led the paper, and the Health & WealthMOD2030 project. RS developed Health & WealthMOD2030 and S.K. developed APPSIM. MC and EC analysed the data. LV worked on the health trend data. RT worked on weighting the data. All authors contributed to data interpretation and drafting the manuscript, as well as approving the final version of the manuscript.

## Pre-publication history

The pre-publication history for this paper can be accessed here:

http://www.biomedcentral.com/1471-2458/14/220/prepub
